# Draft Genome Sequence of Plant Growth-Promoting *Bacillus amyloliquefaciens* Strain W2 Associated with *Crocus sativus* (Saffron)

**DOI:** 10.1128/genomeA.00862-14

**Published:** 2014-09-11

**Authors:** Rikita Gupta, Jyoti Vakhlu, Atima Agarwal, Pravin D. Nilawe

**Affiliations:** a School of Biotechnology, University of Jammu, Jammu, Jammu and Kashmir, India; b Thermo Fisher Scientific, Gurgaon, Haryana, India

## Abstract

*Bacillus* sp. strain W2 is a plant growth-promoting rhizobacterium isolated from saffron fields of Kashmir, India. Here, we report the draft genome sequence (3.9 Mb) of *Bacillus amyloliquefaciens* strain W2 having 65 contigs (3, 997, 511 bp), 4,163 coding sequences, and an average 46.45% GC content. Despite the 99% identity of the 16S rRNA gene with that of *Bacillus amyloliquefaciens* subsp. *plantarum* FZB42, the genome comparison revealed that only 48.7% of the W2 genome has homology with that of FZB42.

## GENOME ANNOUNCEMENT

Plant growth-promoting rhizobacteria (PGPR) are profitable for plant growth and disease control (1). Members of the genus *Bacillus* are known PGPRs (2, 3). Strain W2 is a plant-associated *Bacillus amyloliquefaciens* isolated from saffron fields of Kashmir, India, and is closely related to the commercially available biofertilizer strain FZB42 (RhizoVital 42) based on 16S rRNA gene sequencing. Subsequent gene analysis will throw more light on the functional differences of the two genomes.

The genomic DNA of *Bacillus amyloliquefaciens* W2 was isolated using a cetyltrimethylammonium bromide-based method and sequenced using Ion PGM 400-bp sequencing chemistry (Invitrogen Bioservices Pvt. Ltd.), and a total of 2,718,703 reads, with an average read length of 321 bp were produced. An assembly generated by MIRA version 3.9.18 (4) resulted in 65 contigs with 101× coverage, in which the largest contig size was 1.037 Mb. The unclosed draft genome sequence of *Bacillus amyloliquefaciens* W2 contains 3,997,511 bp, with an average GC content of 46.45%. The contigs were used for gene prediction and annotation by the NCBI Prokaryotic Genome Automatic Annotation Pipeline (http://www.ncbi.nlm.nih.gov/genome/annotation_prok). The sequence was also submitted to the RAST version 2.0 server (5) for annotation and identification of metabolic pathways.

The genome contains 4,163 coding sequences and encodes for 121 RNAs. The genome is predicted to have genes for bacteriocins, resistance to antibiotics and toxic compounds, and siderophore bacillibactin. It also contains genes for flagellar motility, chemotaxis, and stress response. Prophages were also observed in the genome sequence. In comparison with the most closely related species, i.e., *Bacillus amyloliquefaciens* FZB42, only 1.9 Mb of the draft genome was found to be similar and a large inversion (approximately 1 Mb) was observed in a synteny graph.

### Nucleotide sequence accession number.

This whole-genome shotgun project has been deposited at DDBJ/EMBL/GenBank under the accession number JOKF00000000. The version described in this paper is version JOKF01000000.
